# Prävalenz von Schleimhautläsionen bei Durchführung der transnasalen endoskopischen Dysphagiediagnostik und angelegter Nasogastralsonde

**DOI:** 10.1007/s00106-023-01361-3

**Published:** 2023-10-05

**Authors:** Kira-Milena Heise, Simone Miller, Martin Ptok, Michael Jungheim

**Affiliations:** Klinik und Poliklinik für Phoniatrie und Pädaudiologie, Medizinische Hochschule Hannover, Carl-Neuberg-Str. 1, 30625 Hannover, Deutschland

**Keywords:** Pharynx, Nasenhöhle, Erosion, Blutung, Hämatom, Pharynx, Nasal cavity, Erosion, Bleeding, Hematoma

## Abstract

**Hintergrund:**

Zur Einschätzung des Penetrations- oder Aspirationsrisikos bei Dysphagien hat sich unter anderem die flexible endoskopische Evaluation des Schluckakts (FEES) etabliert. Sie gilt als komplikationsarmes Verfahren, ist jedoch bei angelegter Nasogastralsonde (NGS) erschwert durchführbar und könnte mit einer höheren Rate akzidenteller Schleimhautläsionen einhergehen.

**Zielsetzung:**

In dieser Studie sollte ermittelt werden, ob eine erhöhte Prävalenz von Schleimhautläsionen vorliegt, wenn die FEES bei liegender NGS und damit unter erschwerten Untersuchungsbedingungen durchgeführt wird. Präexistente Läsionen sollten ebenfalls erfasst werden.

**Methodik:**

In einer retrospektiven, monozentrischen Studie wurden insgesamt 918 videodokumentierte FEES-Untersuchungen, die zu diagnostischen Zwecken bei stationären Patient*innen mit angelegter Nasogastralsonde von Januar 2014 bis März 2019 an einer Universitätsklinik durchgeführt wurden, ausgewertet. Erfasst wurden Anzahl und Ausprägungen von Schleimhautläsionen.

**Ergebnisse:**

In keinem der hier analysierten Fälle trat eine endoskopiebedingte Verletzung auf. Bei 48,6 % der analysierten Endoskopien konnten jedoch präexistente Läsionen in Nase und/oder Nasopharynx festgestellt werden, die häufig als Mehrfachverletzungen vorlagen.

**Schlussfolgerung:**

Die Ergebnisse der Studie zeigen, dass die FEES hinsichtlich der Gefahr einer akzidentellen Schleimhautverletzung auch bei in situ befindlicher NGS eine sichere und risikoarme Untersuchungsmethode ist. Bemerkenswert ist jedoch die sehr hohe Zahl präexistenter Schleimhautläsionen, die mit hoher Wahrscheinlichkeit in Zusammenhang mit dem zuvor erfolgten Legen einer NGS stehen. Die Häufigkeit präexistenter Läsionen lässt es ratsam erscheinen, Strategien zur Verletzungsminimierung beim Legen von Nasogastralsonden zu entwickeln.

## Abstract (en)

**Background:**

The flexible endoscopic evaluation of swallowing (FEES) is an established low-risk examination method to assess the risk of penetration or aspiration in patients with dysphagia. FEES might be more difficult or of higher-risk when a nasogastric tube is in place.

**Objective:**

This study aims to identify whether the prevalence of mucosal lesions is higher when the endoscopy is carried out with a nasogastric tube in place. Pre-existing mucosal lesions were also documented.

**Methods:**

In a retrospective, monocentric study, a total of 918 FEES procedures routinely performed in hospitalized patients of a university hospital from January 2014 to March 2019 were evaluated. Mucosal lesions were identified and characterized for descriptive statistics.

**Results:**

In the video material analysed here, no endoscopy-related injuries were identified. However, pre-existing mucosal lesions, which often occurred as multiple lesions, were detected in 48.6% of the endoscopies. Further analysis showed that these pre-existing lesions were not worsened by the endoscopy performed.

**Conclusion:**

The results demonstrate that transnasal flexible endoscopy is a safe, low-risk examination method, even in patients with a nasogastric tube. A very high number of pre-existing mucosal lesions were found, which is probably related to the previous insertion of the nasogastric tube. Due to the high number of pre-existing lesions, strategies should be developed to minimize injuries when placing nasogastric tubes.

Bei der Diagnostik von Schluckstörungen mittels der flexiblen endoskopischen Evaluation des Schluckvorgangs (FEES) wird transnasal ein flexibles Endoskop eingeführt und bis hinter das Velum palatinum vorgeschoben, sodass der Pharynx- und Larynx eingesehen und die Schluckfunktion bei unterschiedlichen Boluskonsistenzen beurteilt werden können [5, 11, 12, 17, 18]. Zu den Risiken der Untersuchung zählen unter anderem Schleimhautläsionen, vasovagale Synkopen, Larynxödeme, Atemwegsverlegungen durch das Endoskop, die Aspiration von mobilisierten Borken aus der Nase oder dem Pharynx sowie allergische Reaktionen bei Verwendung von Lokalanästhetika [10]. Bereits erfolgte repräsentative Datenanalysen zeigen, dass auftretende Komplikationen selten, nicht schwerwiegend und zumeist selbstlimitierend waren, wie beispielsweise ein Würgereiz, ein Anstieg des arteriellen Blutdrucks während der Untersuchung oder eine Epistaxis, deren Häufigkeit mit Werten zwischen 0,1 % und 6 % beziffert wird [2, 3, 9–11, 21, 28].

Bei Patient*innen im stationären Setting eines Krankenhauses der Maximalversorgung bestehen häufig schwerwiegende Vorerkrankungen einschließlich Gerinnungsstörungen und der sich daraus ergebenden Einnahme blutverdünnender Medikamente. Wird bei diesen Patient*innen eine FEES durchgeführt, kann vermutet werden, dass das Risiko auftretender Schleimhautläsionen und Blutungen bei der Untersuchung höher ist als bei Patient*innen ohne entsprechende Medikation. Darüber hinaus wird häufig zur Sicherstellung der Ernährung bereits im Vorfeld eine Nasogastralsonde (NGS) angelegt. Eine sich in situ befindende NGS kann die flexible transnasale Endoskopie zusätzlich erschweren, da zumeist derjenige Nasengang mit der Sonde belegt ist, der die einfachste Passage der Sonde ermöglicht. Somit könnte die FEES-Diagnostik bei liegender NGS zu einer höheren Rate an Verletzungen im Nasen- und Nasenrachenraum führen, als in den bereits vorliegenden Studien berichtet wurde.

Das Risikoprofil der Anlage einer NGS ähnelt dem der FEES, denn Nasogastralsonden werden ebenfalls transnasal eingeführt, passieren dann allerdings den Pharynx und werden bis in den Magen vorgeschoben. Die Platzierung erfolgt in der Regel ohne Sichtkontrolle, deshalb muss nach der Einbringung die korrekte Positionierung klinisch oder radiologisch geprüft werden [29]. Bei der Platzierung der NGS kann es zu Verletzungen der Schleimhaut von Nasenraum, Pharynx, Ösophagus und/oder Magen kommen, die insbesondere bei Koagulationsstörungen und fehlender Sicht auf den Defekt unbemerkt bleiben und zu erheblichen Blutverlusten führen können. Anhand von Autopsieergebnissen berichten Smith et al. [25], dass eine Blutung im Nasopharynx lebensbedrohlich sein kann, wenn z. B. Blutthromben aspiriert werden. Weitere Risiken bei der Anlage von NGS sind Vagusreizungen mit reflektorischer Bradykardie bis hin zu Asystolie oder stressbedingter Tachykardie [14], komplikationsträchtigen Durchbrüchen durch die Schädelbasis [22] oder den Spinalkanal mit Schädigung des Rückenmarks [15] und Perforationen von Ösophagus, Magen, Duodenum oder großer Gefäße wie der V. jugularis [26]. Durch Fehlpositionierung, Defekte oder Schlaufenbildung der Sonde kann es zur Verletzung von Trachea, Larynx oder Lunge mit lebensbedrohlichen Komplikationen, wie beispielsweise Mediastinitis, Aspirationspneumonie, Pneumothorax oder Asphyxie durch mechanische Verlegung der Atemwege kommen [4, 13, 16, 25, 26]. Durch engen Kontakt der Sonde zur Mukosa des Pharynx und des Ösophagus können sich Erosionen, Ödeme, Hämatome, Dekubiti, Ulzerationen und Nekrosen entwickeln [4, 6, 20]. Das lebensbedrohliche „nasogastric tube syndrome“ entsteht durch Druck und Reibung der Sonde an Strukturen der postkrikoidalen Region, wobei es in seltenen Fällen zu einer beidseitigen Lähmung der Stimmlippen mit Verschluss der oberen Atemwege kommen kann [1, 7, 23]. Das Verletzungsrisiko für die Mukosa des Nasen- und Nasenrachenraums durch die Anlage einer NGS wurde bislang nicht an repräsentativen Datenmengen evaluiert.

Nachdem in bisher durchgeführten Studien nur ein geringes Komplikationsrisiko für Schleimhautverletzungen bei der Durchführung von endoskopischen Schluckuntersuchungen festgestellt wurde, war es Ziel dieser Studie, anhand von Videomaterial:

1. zu überprüfen, ob sich das Risiko für Schleimhautverletzungen erhöht, wenn die Untersuchungsbedingungen für eine FEES aufgrund schwerwiegender Vorerkrankungen und angelegter NGS eingeschränkt sind,
2. durch die FEES generierte sowie eventuell vorbestehende Mukosaläsionen, die durch die Anlage von Nasogastralsonden auftreten können, zu identifizieren, zu klassifizieren und zu quantifizieren und
3. zu überprüfen, ob es einen Zusammenhang zwischen der die Dysphagie auslösenden Grunderkrankung und der Läsionsausprägung oder -häufigkeit gibt.

## Methoden

### Studientyp

Es wurde eine retrospektive Analyse von videoendoskopischen Schluckuntersuchungen durchgeführt. Die Auswertung des Datenmaterials wurde von der lokalen Ethikkommission genehmigt (8096_BO_K_2018).

### Probanden

Es wurden Videosequenzen stationär behandelter Patient*innen ab 18 Jahren retrospektiv analysiert, die von Januar 2014 bis März 2019 im Rahmen der klinischen Routine konsiliarisch zur Dysphagiediagnostik vorgestellt wurden. Eingeschlossen wurden Patient*innen, die in die Nutzung ihrer Daten zu wissenschaftlichen Zwecken eingewilligt hatten und zum Untersuchungszeitpunkt über eine liegende NGS verfügten.

Bei den Verlaufskontrollen binnen 7 Tagen wurden die Dokumentationen eines Patienten/einer Patientin nur dann eingeschlossen, wenn intraindividuell neue Läsionen im Vergleich zur vorherigen Untersuchung aufgetreten waren. Wurden bei der Erstuntersuchung z. B. Läsionen an der Rachenhinterwand festgestellt, wurden diese bereits dokumentierten Befunde bei Kontrolluntersuchungen innerhalb von 7 Tagen nicht erneut erfasst. Waren bei den Verlaufsuntersuchungen jedoch Läsionen in anderer Lokalisation beobachtet worden, wurden diese als neue Läsion betrachtet.

Ausgeschlossen wurden Patient*innen, die nicht in die Nutzung ihrer Daten zu wissenschaftlichen Zwecken eingewilligt hatten.

### Auswertung der FEES-Untersuchungen

Die FEES wurde von Untersucher*innen durchgeführt, die bereits jeweils mindestens 500 FEES-Diagnostiken vorgenommen hatten. Nach erfolgter Aufklärung der Patient*innen wurde das Endoskop (Fa. Karl Storz SE & Co. KG, Tuttlingen, Deutschland, 11101RP2 Rhino-Pharyngo-Laryngo-Fiberskop, distales Ende Ø 3,5 mm) unter Sicht durch einen Nasengang eingeführt. Innerhalb der Nasenhöhle wurde die Mukosa auf Erosionen, profuse Blutungen und Hämatome untersucht und die Anzahl vorhandener Läsionen erfasst. Bei Vorschub des Endoskops in den Mesopharynx konnte die Rachenhinterwand inspiziert und auf Verletzungen untersucht werden. Nach der Untersuchung anatomischer Strukturen und funktioneller Abläufe des Schluckens von Boli verschiedener Konsistenz wurde das Endoskop vorsichtig unter Sicht zurückgezogen und die Videoaufnahme beendet.

### Untersuchungsregionen und Messgrößen

Die Patient*innendaten wurden anonymisiert. Es wurden die Angaben zu Geschlecht, Alter sowie der Untersuchungszeitpunkt aus dem schriftlichen Konsil ermittelt. Um Zusammenhänge mit Grunderkrankungen aufzeigen zu können, wurden die Patient*innen in folgende Gruppen eingeteilt:

- Gruppe 1 Apoplex
- Gruppe 2 neurodegenerative Erkrankung (M. Parkinson, multiple Sklerose [MS], amyotrophe Lateralsklerose [ALS])
- Gruppe 3 akute entzündliche intrakranielle Erkrankungen (Meningitis, Enzephalitis, Hirnabszess)
- Gruppe 4 Tumorresektion Kopf/Hals
- Gruppe 5 Hirntumor
- Gruppe 6 Schädel-Hirn-Trauma
- Gruppe 7 sonstige Erkrankungen (Sepsis, Intubations- oder Narkosefolgen, idiopathische Dysphagie, keine Angabe der Grunderkrankung)

Zudem wurden aus den Videos folgende Parameter ermittelt:

Anzahl der Läsionen der Nasenhöhle und Rachenhinterwand jeweils beim Einführen und Zurückziehen des Endoskops.

- 0 = keine Läsion
- 1 = eine Läsion
- 2 = zwei Läsionen
- >2 = mehr als zwei Läsionen

**Figure 1:**
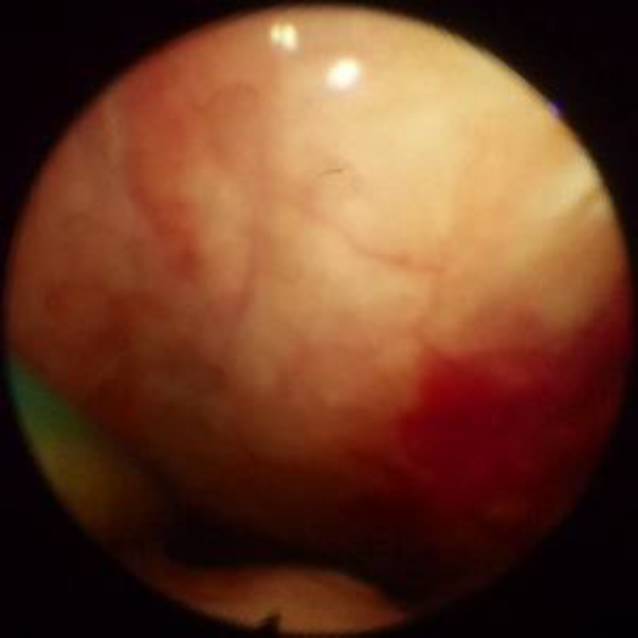

**Figure 2:**
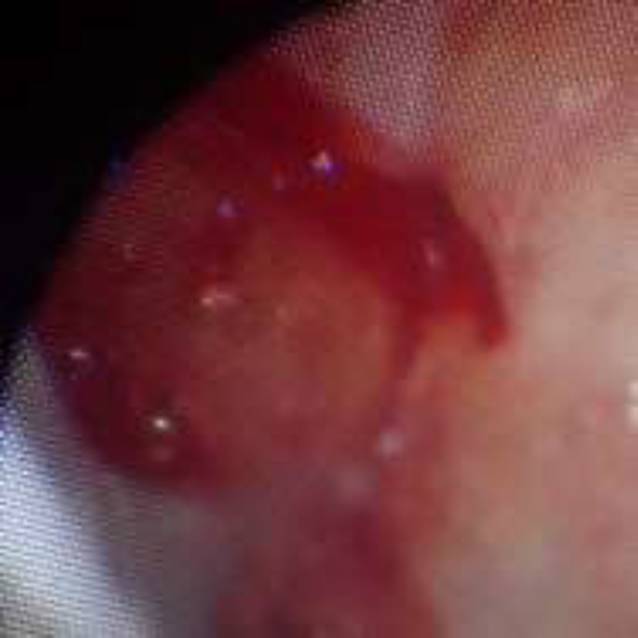

**Figure 3:**
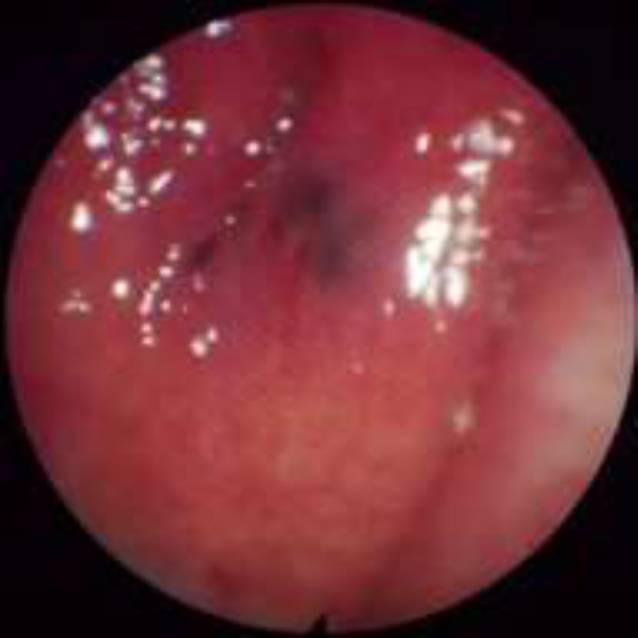

Die Art der Läsion wurde differenziert in *Erosion* (Abb. 1), *Blutung* (Abb. 2) *oder Hämatom* (Abb. 3)**.**

Zudem wurde vermerkt, ob sich das Endoskop im selben Nasengang wie die nasogastrale Sonde befand oder nicht.

### Auswertung und Statistik

Die Auswertung und Darstellung der Daten erfolgten mit der Software Office Professional Plus Excel, Version 2016, Fa. Microsoft Corporation, Redmond, WA, USA.

## Ergebnisse

Insgesamt standen 3993 Untersuchungsbefunde zur Verfügung, von denen 918 die Einschlusskriterien erfüllten. Die Befunde stammten von 737 Patient*innen mit einem Altersdurchschnitt von 66,5 Jahren, außerdem lagen 181 Verlaufskontrollen vor. Von Patienten (Durchschnittsalter 66 Jahre, *n* = 918) stammten 559 Befunde und von Patientinnen (Durchschnittsalter 67,8 Jahre, *n* = 918) 359.

**Figure 4:**
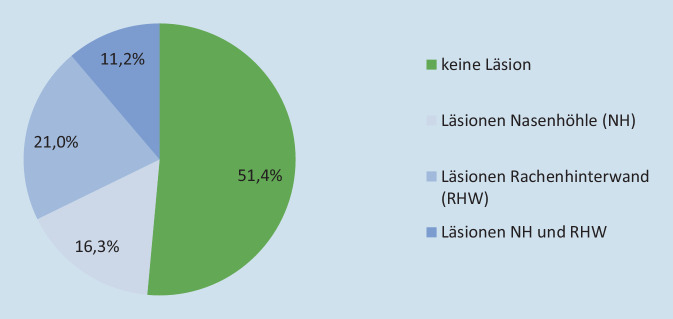

Insgesamt wurden bei 48,6 % der FEES-Untersuchungen unterschiedliche Arten von Verletzungen festgestellt (*n* = 446). Bei 150 Patient*innen bestanden Verletzungen in der Nasenhöhle, 193 Patient*innen wiesen Läsionen an der Rachenhinterwand auf, und bei 103 Patient*innen wurden Verletzungen in beiden Regionen beobachtet (Abb. 4). Zu Mehrfachverletzungen kam es in 37,3 % der Fälle (*n* = 342).

### Läsionen durch transnasale Endoskopie

Es wurden weder in der Nasenhöhle noch an der Rachenhinterwand Läsionen festgestellt, die durch die transnasale Endoskopie hervorgerufen oder durch diese verschlechtert wurden.

### Vorbestehende Läsionen in der Nasenhöhle

Bei 253 von insgesamt 918 Untersuchungsbefunden lagen Verletzungen der Mukosa in der Nasenhöhle vor. Bezogen auf das gesamte Patientenkollektiv entspricht dies einem Anteil von 27,6 %.

Bei 71,9 % der 253 Patient*innen wurden Läsionen in dem Nasengang gefunden, der zum Zeitpunkt der Endoskopie nicht mit einer NGS belegt war.

**Figure 5:**
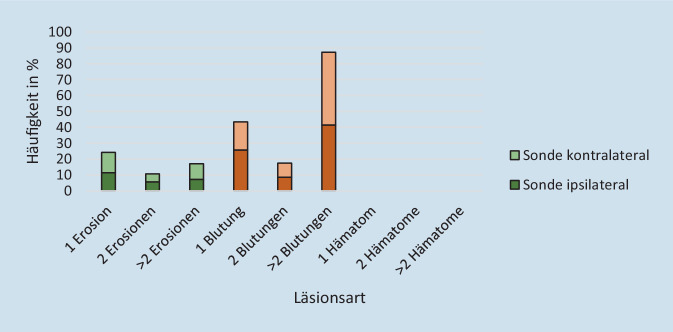

Betrachtet man die Art der dokumentierten Verletzungen insgesamt, so zeigten sich am häufigsten Blutungen (insgesamt 72,3 %) gefolgt von Erosionen der Mukosa (insgesamt 27,7 %). Hämatome wurden nicht identifiziert (0,0 %). Mehrfachverletzungen traten bei 69,2 % der Untersuchungen auf. Die Verteilung der Läsionsarten ist in Abb. 5 dargestellt.

### Läsionen an der Rachenhinterwand in Zusammenhang mit der NGS

Bei 296 von 918 Untersuchungsbefunden wurden Verletzungen der Rachenhinterwand gefunden. Die Rachenhinterwand ist mit einem Anteil von 32,2 % etwas häufiger von Verletzungen betroffen als die Nasenhöhle. Neben einem eher ausgewogenen Verhältnis von Blutungen und Erosionen wurden auch Hämatome festgestellt.

**Figure 6:**
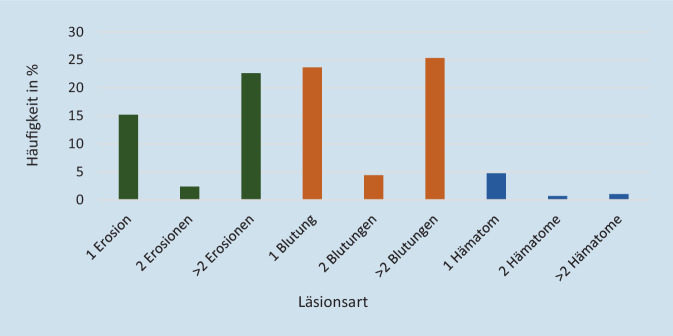

An der Rachenhinterwand zeigten sich weniger Blutungen als in der Nasenhöhle, dafür wurden neben Erosionen auch Hämatome beobachtet. Die Verteilung von Erosionen und Blutungen war relativ ausgewogen. Mehrfachverletzungen traten hier zu 56,4 % auf (Abb. 6).

### Läsionen in Abhängigkeit von der Grunderkrankung

**Figure 7:**
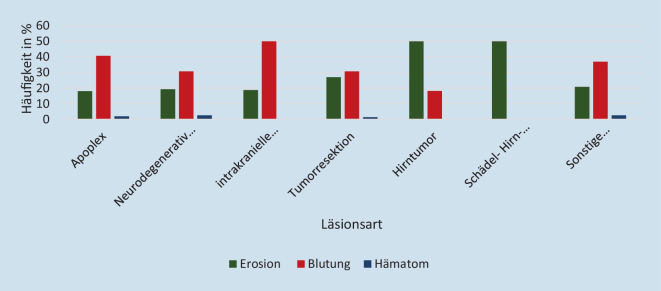

Die Verteilung der Grunderkrankungen des Kollektivs ist in Tab. 1 dargestellt. Betrachtet man die Läsionshäufigkeit in Bezug auf die Grunderkrankung, so lässt sich erkennen, dass in jeder Erkrankungsgruppe bei etwa 60 % der Patient*innen Verletzungen feststellbar waren. Von den 2 Patienten mit Schädel-Hirn-Trauma wies nur ein Patient eine Läsion auf (Abb. 7).

**Table 1:** 

Grunderkrankung	Anzahl	Prozent (%)
Apoplex	367	40,0
Neurodegenerative Erkrankungen	78	8,5
Intrakranielle Entzündungen	16	1,7
Tumorresektion Kopf/Hals	78	8,5
Hirntumor	22	2,4
Schädel-Hirn-Trauma	2	0,2
Sonstige Erkrankungen	355	38,7

## Diskussion

Das Ziel dieser Studie war, bei Dysphagiepatient*innen mit liegender NGS das Risiko für akzidentelle endoskopiebedingte Verletzungen der Mukosa des Nasen- und Nasenrachenraums zu evaluieren sowie präexistente Läsionen festzustellen.

In keiner der in dieser Studie analysierten FEES-Befunde wurden Läsionen durch die Endoskopie verursacht oder durch diese verschlechtert. Die FEES kann daher als risikoarmes Verfahren bestätigt werden, auch wenn die Diagnostik bei platzierter NGS erfolgen muss oder schwerwiegende Vorerkrankungen bestehen. Dies ist sicherlich auch darauf zurückzuführen, dass die Untersuchung fortlaufend unter Sicht erfolgte. So war es den Untersuchenden möglich, durch die Einsehbarkeit der Nasengänge den anatomisch günstigsten Passageweg zu ermitteln, ohne Läsionen durch blinde Tastversuche zu setzen, wie dies bei der Anlage von NGS erfolgen kann. Aufgrund des geringen Durchmessers des Endoskops (Ø 3,5 mm) konnte die Optik auch an der NGS vorbeigeführt werden, wenn dieser Nasengang am besten passierbar erschien. Das Vorgehen unter Sicht hat hier ebenfalls eine Schonung der Schleimhäute bewirkt.

In vorangegangenen Studien, in denen die FEES ebenfalls hinsichtlich ihrer Risiken untersucht wurde, sind höhere Komplikationsraten beobachtet worden. Mit Raten von 0,6–6 % war eine Epistaxis am häufigsten [3, 9, 11, 21, 28]. In der hier durchgeführten Studie lag ein deutlich geringeres Risiko für diese Komplikation vor (0,0 %). Dies ist wahrscheinlich auch auf die Erfahrung der Untersucher*innen zurückzuführen, die mit jeweils mindestens 500 durchgeführten FEES-Untersuchungen als hoch eingeschätzt werden kann. Obwohl Dziewas et al. [11] keinen direkten Zusammenhang zwischen der Erfahrung des Untersuchenden und der Komplikationsrate gefunden haben, sollte in einer zukünftigen Studie die individuelle Komplikationsrate der ersten 50–100 FEES-Untersuchungen des Untersuchenden evaluiert werden.

Die Ursache der präexistenten traumatischen Schleimhautläsionen kann anhand der vorliegenden Patientendaten nicht genau belegt werden. Allerdings würde eine endonasale Absaugung von Sekret nicht zu Läsionen an der Rachenhinterwand führen. Außerdem sind transnasale endotracheale Intubationen aufgrund des Krankheitsprofils der untersuchten Patient*innen als Ursache in den meisten Fällen auszuschließen, da diese häufig bei kieferchirurgischen oder HNO-ärztlichen Eingriffen im Mund- oder Pharynxbereich erfolgen [8]. Somit ist mit hoher Wahrscheinlichkeit davon auszugehen, dass die Läsionen auf die Anlage der Nasogastralsonde zurückzuführen sind, da dieser Eingriff bei allen Patient*innen gleichermaßen erfolgt ist.

Untersuchungen an größeren Patientenkollektiven zum Verletzungsrisiko bei der Anlage von Nasogastralsonden lagen bisher noch nicht vor. Entsprechend den hier erhobenen Daten birgt die Anlage nasogastraler Sonden im Gegensatz zur Durchführung der FEES ein relevantes Verletzungspotenzial für die Mukosa der Nase und des Rachenraums. In der Nasenhöhle hatten 27,6 % der Patienten Schleimhautläsionen, hier überwogen profuse Blutungen. Diese tieferen Gewebeschäden können durch eine Traumatisierung der Mukosa z. B. mit der Sondenspitze entstehen. Inwiefern ebenfalls beobachtete Erosionen abheilenden, ursprünglich tieferen Defekten entsprechen, kann hier nicht sicher geklärt werden. Darüber hinaus wurden 71,9 % der Verletzungen in dem Nasengang festgestellt, in dem zum Untersuchungszeitpunkt keine NGS platziert war. Frustrane Anlageversuche oder Sondenwechsel mit Wechsel des Nasengangs könnten hierfür ursächlich sein.

Aufgrund der häufig beobachteten Hämatombildung und des frequenten Auftretens von Blutungen ist die Rachenhinterwand im Vergleich zur Nasenhöhle noch anfälliger für Verletzungen durch nasogastrale Sonden, hiervon waren 32,2 % der untersuchten Patient*innen betroffen. Die Verletzungen waren nahezu ausschließlich fleckförmig und lagen auf Höhe der Choanen. Das Läsionsmuster weist darauf hin, dass die Sondenspitze nicht den anatomischen Verhältnissen entsprechend im Bogen aus dem Nasengang kommend durch den Nasopharynx in den Mesopharynx geglitten ist, sondern gegen die Rachenhinterwand geführt wurde. Ein forcierter Vorschub der Sonde gegen den Widerstand der Rachenhinterwand, eine falsch gewählte Vorschubrichtung, mangelnde Compliance oder ungünstige Lagerung des Patienten könnten die Schleimhautläsionen verursacht haben [29]. Ein weiterer verkomplizierender Aspekt ist die fehlende Kontrolle über die Sondenspitze bei der Anlage der NGS. Die NGS kann im Gegensatz zu flexiblen Endoskopen nicht vom Behandelnden abgewinkelt und den anatomischen Verhältnissen folgend vorsichtig vorgeschoben werden, sondern kann nur tastend eingebracht werden, um den Nasopharynx und die Engstelle des velopharyngealen Abschlusses zu überwinden. Neben diesen Faktoren könnte die bei Patient*innen mit Dysphagie anzunehmende Dyskoordination der am Schluckvorgang beteiligten Muskulatur und das mögliche Vorhandensein von Sensibilitätsstörungen [24] zu einem erschwerten Vorschub der NGS und zu Abwehrverhalten führen. Werden Läsionen gesetzt, kann es insbesondere bei Vorliegen einer Antikoagulation zu nicht unerheblichen Blutverlusten kommen. Fließt das Blut nicht aus der Nase, sondern in den Rachen ab, wird die Blutung unter Umständen erst spät bemerkt. Ein anderes Risiko geht von aspiriertem geronnenem Blut aus, das zu einer Beeinträchtigung der Lungenfunktion mit potenziell letalem Ausgang führen kann [25].

Bei zwei Patienten wurde in dieser Studie eine Fehlpositionierung der Sonde in der Trachea als Zufallsbefund erkannt. Es kann dadurch zu Verletzungen der Trachea, des Larynx oder der Lunge kommen. Wird Nahrung über die Sonde transportiert, gelangt diese nicht wie erwünscht in den Magen, sondern direkt in die Lunge und kann dort eine Aspirationspneumonie verursachen. Sparks et al. [27] berichten in ihrem Review, dass es bei 1,9 % der 9900 analysierten Sondenanlagen zu einer Fehlpositionierung in den Atemwegen kam, bei 5 Patienten führte ein resultierender Pneumothorax zum Tod.

Trotz der Schwere möglicher Komplikationen bei der Anlage von NGS erfolgt diese standardmäßig „blind“, also ohne visuelle Kontrolle des Sondenvorschubs [4, 19, 25]. Li et al. [19] haben in Hinblick auf die Problematik von Fehllagen nasogastraler Sonden den Einsatz eines fiberoptischen Systems zur Anlage einer NGS an einem Testphantom untersucht. Sie stellten fest, dass die visuell unterstützte Sondenanlage zeitlich effizienter ist als die „blinde“ Insertion und Fehllagen vermieden werden konnten. Das zusätzlich untersuchte Auftreten von Verletzungen der Mukosa des Testkörpers konnte um mehr als die Hälfte reduziert werden.

Auch die Ergebnisse der hier durchgeführten Studie legen nahe, dass eine die Sondenanlage begleitende transnasale Endoskopie zu empfehlen ist. Auf diese Weise können anatomische Strukturen inspiziert werden, und ein Anlageversuch durch einen verlegten oder sehr engen Nasengang kann, ebenso wie eine Verletzung der Rachenhinterwand, unter Umständen vermieden werden. Ein kombiniertes Vorgehen würde auf diese Weise zur Erhöhung der Patientensicherheit beitragen.

Ein Zusammenhang zwischen bestimmten Grunderkrankungen und dem Auftreten von Schleimhautläsionen in der Nase oder im Nasopharynx konnte nicht festgestellt werden. Die Läsionen waren gleichmäßig über alle Diagnosegruppen verteilt und lagen jeweils bei etwa 60 % der Patienten vor (Tab. 1). Ein Einfluss der Grunderkrankung auf die Läsionshäufigkeit oder das Verletzungsmuster kann folglich hier nicht nachgewiesen werden.

## Fazit für die Praxis

- Auch unter schwierigen Untersuchungsbedingungen handelt es sich bei der FEES um ein komplikationsarmes Verfahren.
- Präexistente Läsionen erhöhten bei erfahrenen Untersuchenden das Risiko für zusätzliche Schädigungen nicht.
- Bei annähernd 60 % der untersuchten Patienten lagen Verletzungen der Mukosa der Nase und/oder des Rachenraums vor, die wahrscheinlich auf die zuvor erfolgte Anlage nasogastraler Sonden zurückgeführt werden können.
- Die hohe Prävalenz von Schleimhautläsionen in Zusammenhang mit platzierter NGS zeigt die Notwendigkeit auf, neue Methoden zum sicheren Einbringen der Sonden zu entwickeln.
